# Women Doctors and the London County Council

**Published:** 1914-04-25

**Authors:** Binnie Dunlop

**Affiliations:** Alexandra Court, S.W.


### Women Doctors and the London County Council.

*To the Editor of* THE HOSPITAL.

SIR,—"Interested" says that no public authority should employ the sort of woman who would marry with the deliberate intention of avoiding maternity. Is your correspondent really unaware that the practice of contraception is now almost universal among married people of the educated classes, including the clergy? Indeed, the extending of this knowledge to the wage-earners, few of whom can possibly do justice to more than two children, is—on humanitarian, eugenic, and anti-socialistic grounds—by far the most urgently needed reform of the day.

But whatever the reason for the London County Council's decision to continue the policy of requiring women doctors to resign their appointments when they marry it is an unjustifiable interference with their personal liberty.—Yours respectfully,

BINNIE DUNLOP, M.B., Ch.B.

Alexandra Court, S.W.

